# A broken heart due to obsessive-compulsive disorder (OCD): a case report of Takotsubo Syndrome in a woman with untreated OCD

**DOI:** 10.1192/j.eurpsy.2025.1721

**Published:** 2025-08-26

**Authors:** E. Serna Crespo, M. Carrasco Álvarez, J. Hidalgo-Lopez, R. De Hita Santillana

**Affiliations:** 1 Psychiatric Trainee; 2 University Hospital Jose Germain, Leganes, Spain

## Abstract

**Introduction:**

Takotsubo cardiomyopathy, or broken heart syndrome, is a rare condition characterized by a temporary decrease in the left ventricle ejection fraction. Takotsubo was first described in Japan in 1990. 90% of cases occur in women over 67 years of age. Although the clinical presentation is similar to acute myocardial infarction, normal coronary arteries are usually detected upon cardiac catheterization. The etiology is not defined yet, but all the studies conclude that physical or emotional stress generates a release of catecholamines that produces a transient left ventricular apical dysfunction.

Obsessive-compulsive disorder (OCD) is characterized by recurrent intrusive thoughts, images or impulses. These obsessions provoke distress (typically anxiety) and compulsions which can be described as repetitive behavioral or mental acts that the person feels compelled to perform. If they don’t perform the compulsions, the anxiety rises.

**Objectives:**

To highlight the importance of treating mental illness to promote health in a multidimensional way.

**Methods:**

We review the currently available literature on Takotsubo cardiomyopathy and its emotional triggers. We also study OCD as a stress factor and the characteristics of this pathology. Finally, we present a case report of a 37-year-old woman, with no previous contact with Mental Health Services. She had developed since the Covid 19 pandemic a severe obsessive-compulsive disorder, which was neither diagnosed nor treated. In the 36th week of pregnancy, after several days without leaving her home due to the limitations of her OCD, she went out into the street, which caused her considerable stress. The next day she suffered from Takotsubo cardiomyopathy or broken heart syndrome, with added analytical signs of preeclampsia, for which a cesarean section was also performed.

**Results:**

In this case, the patient had been suffering from severe obsessive-compulsive symptomatology for approximately 4 years, which had worsened during pregnancy. This caused her a significant level of stress and anxiety, which in the absence of psychiatric treatment, could have endangered her life and that of the baby.

**Conclusions:**

Mental illness tends to have serious medical consequences for patients, that could be prevented with proper treatment of their psychiatric pathology.

**Disclosure of Interest:**

None Declared

